# The public mental representations of deepfake technology: An in-depth qualitative exploration through Quora text data analysis

**DOI:** 10.1371/journal.pone.0313605

**Published:** 2024-12-30

**Authors:** Barbara Caci, Giulia Giordano, Marianna Alesi, Ambra Gentile, Chiara Agnello, Liliana Lo Presti, Marco La Cascia, Sonia Ingoglia, Cristiano Inguglia, Alice Volpes, Dario Monzani

**Affiliations:** 1 Department of Psychology, Educational Sciences and Human Movement, University of Palermo, Palermo, Italy; 2 Department of Engineering, University of Palermo, Palermo, Italy; 3 Independent Researcher, Padova, Italy; 4 Applied Research Division for Cognitive and Psychological Science, European Institute of Oncology IRCCS, IEO, Milan, Italy; Universiti Teknikal Malaysia Melaka Fakulti Teknologi Maklumat dan Komunikasi, MALAYSIA

## Abstract

The advent of deepfake technology has raised significant concerns regarding its impact on individuals’ cognitive processes and beliefs, considering the pervasive relationships between technology and human cognition. This study delves into the psychological literature surrounding deepfakes, focusing on people’s public representation of this emerging technology and highlighting prevailing themes, opinions, and emotions. Under the media framing, the theoretical framework is crucial in shaping individuals’ cognitive schemas regarding technology. A qualitative method has been applied to unveil patterns, correlations, and recurring themes of beliefs about the main topic, deepfake, discussed on the forum Quora. The final extracted text corpus consisted of 166 answers to 17 questions. Analysis results highlighted the 20 most prevalent critical lemmas, and deepfake was the main one. Moreover, co-occurrence analysis identified words frequently appearing with the lemma deepfake, including video, create, and artificial intelligence—finally, thematic analysis identified eight main themes within the deepfake corpus. Cognitive processes rely on critical thinking skills in detecting anomalies in fake videos or discerning between the negative and positive impacts of deepfakes from an ethical point of view. Moreover, people adapt their beliefs and mental schemas concerning the representation of technology. Future studies should explore the role of media literacy in helping individuals to identify deepfake content since people may not be familiar with the concept of deepfakes or may not fully understand the negative or positive implications. Increased awareness and understanding of technology can empower individuals to evaluate critically the media related to Artificial Intelligence.

## Introduction

Deepfake technology, powered by Artificial Intelligence (AI) techniques associated with advanced machine learning algorithms [1], has ushered in a new era of realistic and sophisticated synthetic media by manipulating images, video, and audio content. This technology produces altered media of remarkable realism, often generating deceptive content indistinguishable from their authentic counterparts [2]. Indeed, deepfakes create a “simulation of the speaker in a hyper-realistic video” [3; p.16], representing people doing and saying things that have ever actually happened [4] by mimicking people’s facial expressions and voice modulations [5]. From a technical point of view, deepfake generation primarily relies on deep learning architectures, specifically Variational Autoencoders (VAEs) or Generative Adversarial Networks (GANs). The former were proposed by [6] employing probabilistic models to encode and decode data, offering a different approach to deepfake synthesis. The latter was introduced by [7] and comprised a generator and a discriminator network involved in an adversarial training process, resulting in highly realistic synthetic media. Deepfakes have become widely associated with applying deep learning techniques for face replacement [8], including facial reenactment and lip-syncing, all geared toward creating highly realistic videos. For instance, Deep Video Portraits [9] employ neural networks to transfer facial expressions from a prior source to another, producing lifelike visual outputs. Lip syncing, a critical component in audio-visual synchronization, is achieved through techniques like SyncNet [10] to enhance the inner coherence of the generated content. Recently, deepfake applications have gained significant popularity and have also been positively employed in various industries, including movies, gaming, social media, education, healthcare, material science, fashion, and e-commerce [11] For instance, apps such as Deepware Scanner, Descript Overdub, RefaceApp, Avatarify, and Lil Miquela have been identified as noticeable for their distinctive features and varied functionalities. The proliferation of deepfake technology is receiving increasing attention in academia, including social sciences, humanities, computer science, political sciences, and law. Even though scholarly research is still lacking in integrating different perspectives on mechanisms involved in creating, consuming, and disseminating deepfakes [12]. The predominant matters of recent literature have been on deepfake negative implications and risks for media exposition, ethical concerns, and an overall necessity to increase public awareness regarding the potential misuse of AI [11]. There is a growing concern that users might soon be unable to discern content generated by machines from authentic ones, making them vulnerable to disinformation campaigns [13]. Experts worry about the malicious use of deepfakes in damaging societies [14] Indeed, the use of deepfakes for spiteful purposes, such as nonconsensual pornography [15,16] (and political disinformation, undermines trust in institutions, media, online communication platforms, and societal values [17,18]. Some studies reported that exposure and sharing of deepfake videos could cause skepticism in considering new social media content [19–22] (Ahmed, 2021a; 2021b; 2023). In the political field, several factors heighten the threat of deepfake disinformation, such as human tendencies to be drawn to shocking content often present in deepfakes, contributing to broader audiences and facilitating dissemination [23]. Examples of deepfakes used for political propaganda were the fake video in which Obama swore at Donald Trump during an announcement in public [22] and the more recent fake video depicting Ukrainian President Volodymyr Zelenskyy issuing orders for his soldiers to lay down their weapons and cease fighting against Russia [24]. A study [8] highlighted university students’ main concerns regarding the spread of disinformation since malicious utilization of deepfake videos has been employed to create convincing fake news or manipulate public opinion. Other potential risks rely on privacy concerns since deepfake technology allows fake but realistic videos or audio recordings to be created without individual’s content, raising legal and ethical issues founding that privacy protection is the most sensitive factor [25,26]. Ethical concerns related to deepfakes include privacy protection, traceability, and informed question as factors that influence ethical acceptability directly and social acceptance indirectly. In contrast, perceived enjoyment directly affects social acceptance of deepfakes by weakening the effect on ethical acceptability on social acceptance [26]. Also, the use of deepfakes for entertainment purposes could raise concerns about manipulating hyper-realistic digital representations of individuals’ images and voices, which should be considered a fundamental moral right [27].

Scholars in cognitive psychology discussed individuals’ mental processes underlying the fruition and detection of real or deepfake video. The mixture of realistic audio and visual cues could cause people to use their realism heuristic [28,29], in which visual cues precede signals from other senses in prompting responses and information storage [30,31] so creating even false memories [32,33]. Using heuristics facilitates cognitive efficiency since people select specific sensory inputs to process, disregarding other stimuli. Therefore, the level of attention paid affects the amount of information assimilated and processed [21] to prevent an overwhelming accumulation of information which could result in cognitive overload and, consequently, failure to comprehend and assimilate the content of the messages [34]. Individuals perceive audio and images as more closely mirroring the real world than text [22]. Within the metacognitive experience, fluency is central to comprehending why people believe false information. As suggested in [35], fluency implies that humans are more inclined to perceive messages as valid if they are familiar with them. This sense of familiarity causes a truthiness effect—i.e., a perceptual flow that facilitates easier assimilation of material, rendering it more believable [36]. Activated when images and audiovisual content prove more accessible and comprehensible than written texts, as delineated by [37], this metacognitive experience becomes instrumental in shaping people’s cognitive task responses, notably in elaborating novel information. The technical realism of deepfake videos, mainly when depicting famous or widely recognized individuals, potentially compounds the preexisting concern that fluency can be achieved by invoking familiarity, regardless of the video’s content accuracy. A third-person perception bias might also arise, and people are more inclined to believe that deepfakes affect others more than themselves and that they are better at distinguishing deepfakes than others. The third-person perception bias is less effective in children or adolescents due to their levels of cognitive development. However, it is more pronounced among adults with higher cognitive skills who are more skeptical of social media news and less inclined to evaluate deepfakes as accurate or share them. However, this bias does not necessarily align with people’s ability to distinguish deepfake videos from real ones, as demonstrated by a deepfake detection test [21]. People outperform AI detection systems because of their ability to process faces holistically and visually [38]. Also, people may become excessively vigilant towards possibly manipulated media, overestimating the prevalence of deepfakes [39]. Overconfidence stands out as a prevalent and costly bias in people’s decision-making. When people detect synthetic content, overconfidence could render people susceptible to manipulation. If individuals are confident in their ability to spot a deepfake but cannot, they may inadvertently engage with manipulated content. Moreover, analytic thinking and political interest are related in a positive way to correctly detecting deepfakes and are associated with the ability to discriminate fake news negatively [40]. People with high cognitive levels [21] interacting with people’s cognitive biases on deepfake might exacerbate distrust in news and information disseminated by public figures [11,41]. In essence, trust constitutes an alternative decision-making mode, arising from willingly exposing oneself to vulnerability, acknowledging the inherent risk of betrayal, and giving precedence to someone’s words over other forms of information [13]. Studies have underscored that media literacy education plays a crucial role in empowering individuals to critically evaluate the media they consume, including deepfakes. Interconnected with information and digital literacy, the media literacy aims to equip individualswith the skills to become informed and discerning consumers of media, enabling them to interpret messages and engage meaningfully with media content, even identifying biases [42].

Increased awareness and understanding of technology allow individuals to evaluate the media they consume, improving their ability to recognize and critically evaluate deepfake content [43,44].

Although many studies focused on the above-mentioned negative implications of deepfake, the current literature also reports the potential benefits and positive use cases of deepfake technology [17]. In a case study, deepfake technology was applied in educational settings to create realistic simulations or training scenarios for medical professionals who could practice and refine their skills in a virtual environment [45]. Besides, deepfake technology seems helpful in improving accessibility for individuals with disabilities since it can generate realistic sign language interpretation or assistive technologies for those with speech impairments, but researchers are also discussing potential risks [46]. According to [47], deepfakes could be used for creating simulated learning experiences, allowing students to practice skills in a safe and controlled environment, or for historical reenactments, bringing historical figures to life and allowing students to interact with them so gaining a deeper understanding of historical events. Besides, deepfakes can generate realistic conversations with native speakers, providing students with immersive language learning experiences [48]. For instance, the CereProc group recreated the authentic speech of John Fitzgerald Kennedy in July 1963 about the resolution to end the Cold War, based on previous speeches that he had given (https://www.cereproc.com/en/jfkunsilenced ).

### The present study

The current study aims to delve into the cognitive mental representation in people’s perception of deepfake content, shedding light on the complex relationship between technology and psychological processes. Specifically, we applied thematic analysis using Tlab-10, a computer-assisted qualitative analysis software, to unveil patterns, correlations, and recurring themes of information discussed on Quora, an online forum by Quora Inc., founded in 2009, allowing users to publish questions and answers on specific topics. Questions and answers are grouped by topic, allowing users to vote or comment. Additionally, users can collaborate by modifying questions or answers provided by others. We opted for this platform due to its public nature and unrestricted access to media content. Like social platforms such as Twitter, it offers the advantage of not imposing character limits. Compared to Reddit, Quora has received less criticism for spreading misinformation [49].

The Theoretical and Conceptual Background section (Section 2). The Method section outlines the data collection and analysis procedures (Section 3). Results are subsequently discussed, highlighting prominent themes, and relating them to existing literature on deepfakes and human-computer interaction (Section 4). The Discussion reports the practical implications of the findings, emphasizing the significance of understanding public perceptions of deepfakes for comprehending their cognitive, social, and cultural implications (Section 5). Finally, Strengths and Limitations (Section 6) and Conclusion (Section 7) are reported.

### Theoretical and conceptual background

#### Schema and media framing theory

The well-known schema theory [50–52] in brief affirms that, schemas are organized knowledge stored in the minds that people develop by interacting with the environment through the assimilation process [50,53]. They regard declarative ‐ i.e., knowledge of what is an object, a fact, or an event ‐ and procedural knowledge ‐ i.e., knowledge of how to perform an action or behavior–[54]. Then, such cognitive schemas or knowledge units for a subject or event guide individuals to organize and process new information [55]. Every time people face new information acquisition, the perception associated is not solely driven by external stimuli but is shaped by pre-existing knowledge structures [56]. Pre-existing knowledge could be adapted to new information through the accommodation process. Schemas also affect memory since they guide individuals’ ability to pay attention to crucial information for encoding and help comprehend news by integrating it with prior knowledge [56,57]. In this way, the process allows the brain to manage its resources effectively, processing a few items but generating complex responses to them instead of processing a large amount of accessible information superficially [58]. Under schema theory [50–52], individuals could have pre-defined mental schemas or expectations about deepfake videos regarding information exchange. Therefore, schemas play a crucial role in attributing meaning to what happens around us and facilitating the evaluation, processing, and organization of a wide range of new information [55]. In [59], a pioneering exploration of framing is provided, so that media framing research is born. A media frame refers to a stable and socially shared system of categorization that influences how people perceive and behave in social contexts. A *frame* is a cognitive structure that shapes people’s perception and mental representation of events. For instance, texts like newspaper clippings elucidate how primary frameworks function through keying, fabrication, and anchoring processes [59]. In the media framing theory, the active mental process of selecting frames and their outcomes is also emphasized [60]. According to a prominent perspective, framing entails selecting certain features of a perceived reality and highlighting them in a text "in a way as to promote a particular problem definition, causal interpretation, moral evaluation, and treatment recommendation" [60, p. 52]. In other words, framing influences the selection of events for news and how they are represented. By selecting and reporting news, the media leads people to focus on some problems and issues, not others [61]. People’s reactions to framing in communication texts are primarily influenced by the standard schemas already present in their minds, which originate from various sources. For instance, a study underscored how exposure to framing strategies, particularly in the context of political campaign news, could evoke a recall of specific information strategies within individuals, consequently fostering the attribution of cynical motives to political actors [62,63]. This phenomenon embodies what is commonly referred to as a *priming effect*, wherein individuals’ perceptions and evaluations are subtly influenced by the framing of information they encounter [64]. Media framing is central to ongoing research in communication. A *media frame* (i.e., graphical or visual, written or spoken) represents a tool that individuals use to provide context for a topic (i.e., event, issue, or people) which, throughout mediation, can be transmitted. Indeed, the concept of framing is a tool for shaping public discourse, particularly concerning the dissemination of information by media entities [63]. As stated, framing encapsulates a macro-attribute akin to an issue frame, wherein information sources strategically utilize various devices to meld and articulate opinions or preferences about a given situation. These frames may manifest in either a generic or topic-specific manner, thereby influencing the accessibility of information, either by emphasizing message salience or subtly biasing information processing mechanisms [63].

Examining media framing in the coverage of deepfake technology offers valuable insights into the evolving landscape of digital media regulation. By critically analyzing the framing strategies employed in news coverage, researchers can reveal the underlying narratives that shape public perceptions of deepfakes and inform discussions on regulatory interventions in the digital sphere. According to [65], media framing influences and reinforces collective views of reality. Public perceptions of situations or social groups shape the solutions deemed appropriate for addressing societal challenges, and in communication media frames are employed to align individuals with the contextual information included within the frame references, influencing their perceptions and behaviors regarding a specific topic [63]. In recent years, media framing and cognitive schemas have formed beliefs, expectations, and attitudes toward technology [66]. A specific focus is addressed on people’s cognitive ability to create their mental schemas to understand information spread through social media and the subsequent capability to distinguish real news from fake news. Through framing, news media can potentially increase people’s awareness of a particular topic, drawing attention to actions and emphasizing potential solutions related to health and foreign policy [67,68]. Drawing upon expectancy-value theory [69] subsequent research articulated a comprehensive theory of opinion formation, elucidating the intricate interplay between journalistic framing and individual cognitive processes. This theoretical framework integrates the cognitive importance of information accessibility with two additional constructs, namely, availability and applicability [63]. According to this theoretical paradigm, the information presented within issue frames interacts dynamically with individuals’ pre-existing knowledge and beliefs, eliciting accessible considerations that are readily recalled. Consequently, individuals consciously or subconsciously evaluate this information within their existing knowledge structures, shaping their overall attitudes and opinions on a given issue. Under the framework of cognitive psychology, authors examined how the valence-framing effects of information, whether in a positive or negative light, can systematically influence audience reactions. In [70] Levin and colleagues (1998) is proposed that the attribute-framing effect that occurs when positive framing elicits a more favorable response and negative framing leads to a less favorable response. This effect is thought to arise from the underneath psychological processes of information encoding and memory association. Specifically, the negative framing of an attribute can influence how information is encoded, potentially triggering an unfavorable memory association, while positive framing has the opposite effect. Additionally, it is suggested that the different encoding resulting from negative or positive valence frames may cause people to focus on the information differently. Moreover, the relationship between media frames and audience frames can be influenced by various factors, including social-cultural, organizational, individual, or ideological differences related to the issue [66]. Therefore, it is essential to investigate emerging valence frame trends in deepfake YouTube videos and their audiences. This will enable other researchers in the field of deepfake technology to advance their studies.Indeed, audience frames facilitate the processing of issue frames and play a constitutive role in forming opinions and expressions [71].

Thus, journalistic framing is a potent mechanism through which information is disseminated, shaping public discourse and perceptions on various socio-political issues.

In the era of deepfakes, information about the story’s setting, characters, and timeline is twisted, painting a dystopian future picture, and envisioning a society controlled by altered content and disinformation. This usually happens when journalists narrate deepfakes as a politically and socially counterproductive phenomenon [71].

## Method

### Data collection

Data were extracted from Quora.com on November 20, 2023 (see S1 File). Specifically, we used the Quora internal search function to identify all questions related to deepfake by using “deep fake” or “deepfake” as search terms. The search identified 32 questions. We manually filtered these questions based on the following criteria: 1) Relevance: questions must explicitly mention “deep fake” or “deepfake” to ensure they are directly related to our research topic; 2) Content Availability: questions must have at least one answer to be included in the corpus.

This filtering process resulted in the exclusion of 4 questions that did not contain the search terms and 11 questions that did not have any answers, leaving us with 17 relevant questions.

We then used Octoparse software (Octoparse Data Inc) to extract the text and publication date of individual answers to each question. Octoparse is a free web scraping software that takes unstructured data and text from websites and exports them to a structured data file. This focused approach ensured that the corpus consisted solely of questions directly relevant to the topic, thereby strengthening the meaningfulness and relevance of the data collected. By concentrating on questions that explicitly mentioned deepfakes, we aimed to capture answers covering a comprehensive range of perspectives and discussions surrounding this emerging technology. This methodological choice was essential in ensuring that the subsequent analysis accurately reflected public opinions and mental representations of deepfake. The final text corpus consisted of 166 answers to 17 questions. We included all answers to the selected questions, as each was directly relevant to the topic of deepfakes, ensuring that the corpus fully represented the perspectives and discussions related to the subject.

Answers were posted between February 10, 2018, and November 12, 2023. On average, questions received approximately ten answers (modal value = 5; min = 2; max = 24). The length of the answers was heterogeneous, ranging from three-word statements to a paragraph of 1,146 words (Mean = 145.24; SD = 174,60). This variability in answer length contributed to a rich dataset, capturing a wide range of perspectives on the topic of deepfakes. The study collected freely available public data from Internet forums. The data was accessed and analyzed following the platform’s terms of use and all relevant institutional/national regulations. Data use complied with ethical guidelines for internet research [72]. The European Union General Data Protection Regulation 2016/679 allows for the use of anonymous data for research purposes under certain conditions. Since all analyses have been performed on public and anonymized data, no institutional review board approval was required for the use of this database or the completion of this study.

### Data pre-processing

Before analyzing data, the text corpus underwent accurate pre-processing procedures to ensure consistency and accuracy across the dataset. Specifically, manual intervention was undertaken to rectify typographical errors and standardize terminology usage. Notably, terms such as "deep fake" or "deepfake" or “deep fakes” or “deepfakes” were consistently recoded as "deep_fake" to foster uniformity throughout the corpus. Furthermore, terminological standardization was performed to avoid ambiguity and implement more robust analytical processes. Noteworthy efforts included the substitution of abbreviated terms such as "AI" with "artificial_intelligence" and "ML" with "machine_learning," ensuring clarity and precision in subsequent semantic analyses. Naming conventions were applied to ensure consistency in the representation of people within the corpus. Notably, proper nouns and surnames, such as "Queen Elizabeth" or “George Lucas," were formatted uniformly as "queen_elizabeth" and "george_lucas," respectively, facilitating coherent identification and analysis. The corpus was primed for subsequent analysis with enhanced uniformity and accuracy by systematically recording terms and addressing typographical inconsistencies. Finally, the answers’ publication dates were categorized as “Older answers” (i.e., more than three years old) or “Newer answers” (i.e., two years old or less).

### Analysis method

The individual answers were analyzed through T-Lab 10 [73], a computer-assisted qualitative analysis software that included linguistic and statistical tools for text mining and thematic analysis. Upon importing the corpus into T-Lab, each answer underwent an automated segmentation process into elementary contexts, defined as short paragraphs of similar length. This resulted in a total of 566 elementary contexts, which provided a manageable unit for analysis. The coding process involved the identification of significant words and lemmas within the corpus. The coding was performed through automated lemmatization, which reduced words to their base forms, allowing for a more straightforward analysis of frequency and co-occurrence. Lemmatization is the process of reducing words to their base or dictionary form (the lemma), based on their intended meaning in context. For example, the words “creating”, “created”, and “creates” would all be reduced to the lemma "create." This allowed us to group together inflected forms of a word and analyze them as a single item Following our main aim, the analytical process consisted of three main phases:

1. Identification of the most frequent lemmas used in deepfake-related Quora answers. The primary objective of this analysis is twofold: first, to discern the predominant lemmatized forms of words utilized within the deepfake-related Quora corpus, and second, to quantify their respective frequencies of occurrence. Specifically, after lemmatization, we conducted a frequency analysis to determine the most frequently occurring lemmas in the corpus. This analysis was performed automatically by T-Lab, which counted the number of times each lemma appeared across the 566 elementary contexts that made up the corpus. To identify the most used lemmas, we focused on the frequency of each lemma in the corpus. We ranked all lemmas based solely on their occurrence counts, selecting the top 20 lemmas that were most frequently used in discussions related to deepfakes.
2. Co-occurrence analysis through “word association”. Through this analysis, we aimed to identify words frequently appearing with the lemma “deep_fake” within elementary contexts and identify semantic relationships and associations between terms. We utilized T-Lab’s automated tools to calculate co-occurrence frequencies, focusing specifically on the instances where the lemma “deep_fake” co-occurred with other lemmas. The analysis generated tabular and graphical representations that highlighted these co-occurrences, allowing us to visualize the relationships between terms. To quantify the strength of these associations, we employed the Chi-square test as a measure of association. This statistical test helped us determine whether the observed co-occurrences of “deep_fake” with other lemmas were significantly higher than what would be expected by chance. By focusing solely on the co-occurrences of “deep_fake” with other lemmas, we aimed to identify key word pairs that reflect the most salient themes and sentiments in discussions about deepfake technology. Specifically, by utilizing word associations, we construct tabular and graphical representations highlighting the co-occurrence of the lemma “deep_fake” with other words, offering insights into the complex network of relationships it has within the corpus and providing an in-depth understanding of how these terms interrelate in the discussions surrounding deepfake technology.
3. Identification of coherent semantic clusters defined by distinctive word patterns, commonly called themes. This involved conducting Singular Value Decomposition (SVD) and successively hierarchical cluster analysis (i.e., Principal Direction Divisive Partitioning and K-means method) [74,75] SVD serves as a tool for reducing dimensionality, revealing latent dimensions that underlie semantic similarities among words. By applying SVD, we were able to identify patterns in the data that might not be immediately apparent, thus facilitating a more nuanced understanding of the relationships between terms. Subsequently, cluster analysis utilized the outcomes of SVD to pinpoint semantic clusters, or themes, characterized by specific word arrangements. In this step, we assessed the coherence and distinctiveness of the clusters, ensuring that each theme accurately represented a significant aspect of the data. This methodological approach adheres to a ’bottom-up’ methodology rooted in induction, wherein the themes extracted are closely tied to the empirical data. This inductive approach allowed us to remain grounded in the actual responses from participants, ensuring that the themes were reflective of the public discourse surrounding deepfake technology. By closely linking the themes to the data, we aimed to capture the complexities and nuances of public perceptions.
4. Testing the associations of thematic clusters with answers’ publication data. We analyzed the relationship between thematic clusters and answer publication dates using a Chi-square test and a residual analysis. The residual analysis helped identify significant associations between themes and publication dates by considering: a) standardized residuals greater than 1.96, indicating that the number of observed values was significantly greater than expected, and b) residuals below −1.96, indicating that the number of observed values was significantly less than expected.

## Results

### Key Lemmas analysis: Unveiling trends in deepfake discourse

In examining the deepfake corpus, we focused on analyzing the 20 most prevalent critical lemmas aimed at discerning predominant trends within the discourse. As shown in Table 1, the frequencies attributed to each lemma provide significant insights into the main aspects that users primarily focus on while discussing deepfake technology on Quora.

**Table 1:** The most frequently used lemma.

Lemma	Frequency	Lemma	Frequency
deep_fake	515	person	80
video	293	look	79
fake	146	content	70
create	144	audio	60
technology	135	news	56
image	110	good	55
people	98	voice	47
face	92	believe	44
artificial_intelligence	91	app	43
use_to	82	help	43

Unsurprisingly, “deepfake” emerges as the predominant lemma, since it was one of the main keyword we applied on researching inside the Quora community. The high prevalence of “video” underlines the significant emphasis on the visual aspect of deepfake content and its implications within the discussions. At the same time, the frequency of “fake” highlights possible concerns and the general awareness surrounding the potential deceptive nature of deepfake-generated content. Other most frequent lemmas, such as “create” and “technology,” underline the active generation and production of deepfake content, respectively, reflecting an interest in the creative dimensions of this technology and the users’ interest in understanding the underlying technical aspects and advancements associated with deepfake.

### Co-occurrence analysis: Unveiling semantic relationships

In the specific context of our analysis, co-occurrences of words were computed within elementary contexts defined during the corpus importation phase. This robust approach ensures that the evaluation is conducted at granular levels, allowing us to capture the nuanced associations that shape the semantic landscape of the key lemmas in our deepfake corpus. Specifically, we assessed associations of the lemma “deep_fake” with other lemmas (Table 2).

**Table 2:** Word association analysis for “deep_fake” within the Quora corpus.

Lemmas	Coeff.	EC(B)	EC(AB)	χ^2^	*P*
video	.552	207	140	21.216	< .001
create	.470	110	87	32.151	< .001
technology	.470	110	87	32.151	< .001
artificial_intelligence	.415	79	65	27.706	< .001
use_to	.367	67	53	17.915	< .001
content	.343	58	46	15.495	< .001
fake	.342	99	60	1.552	.213
image	.338	85	55	3.848	.050
audio	.315	52	40	11.171	.001
person	.309	65	44	4.819	.028

*Note: Coeff. = value of the coefficient; EC (B) = total number of elementary contexts including the associated lemma (B); EC (AB) = total number of elementary contexts in which lemmas A and B appear together (i.e., co-occurrences). This table displayed only the first ten lemmas B with the higher coefficients*.

The identified word pairs offer significant insights into public perceptions of deepfakes. For instance, the strong association between “deep_fake” and “video” suggests that discussions predominantly focus on the visual implications of this technology. Similarly, the co-occurrence with “create” highlights the perception of deepfakes as tools for content generation, which can be both innovative and concerning. Terms like “artificial_intelligence” and “technology” further emphasize the advanced technological underpinnings of deepfakes, indicating that users are aware of the sophisticated methods involved in their creation. Conversely, the presence of words such as “fake,” “news,” and “manipulation” reflects public anxiety regarding the potential for misinformation and ethical dilemmas associated with deepfake technology. This duality in word associations illustrates the complex nature of public sentiment, where excitement about technological possibilities coexists with significant concerns about trust and authenticity in media.

**Fig 1:**
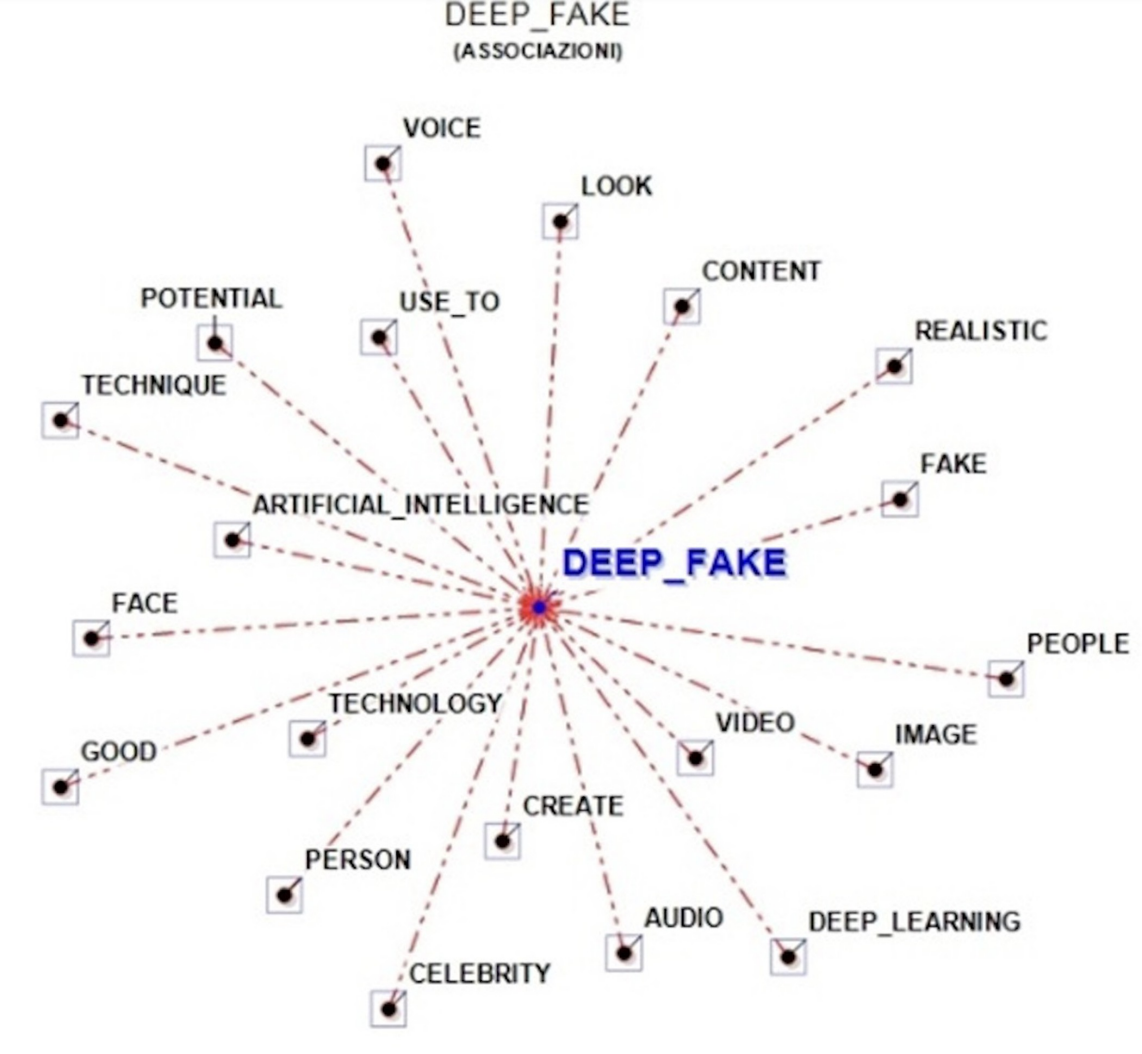
Graphical representation of the word association analysis.

In details, as also shown in Fig 1, users associated with “deep_fake” lemmas related to the creation (i.e., “create”) of synthetic media, mainly “video” or “image” or “audio,” through “artificial_intelligence” and new “technology.” For example, users wrote: “deep_fake ‐ a combination of deep_learning and fake is a term for videos and presentations enhanced by artificial_intelligence to present falsified results. One of the best examples of deep_fake involves videos of celebrities, politicians or others saying or doing things that they never actually said or did.” or “deep_fake can create numerous possibilities and opportunities for all, regardless of who they are and how they interact with the world around them. What is deep_fake technology? Simply put, deep_fake is a technology that easily lets you make and create the realistic-looking digital avatar of any real person.”

### Thematic analysis: Unveiling the discursive threads in the quora corpus on deepfake

As a final analysis output, we identified eight main themes within the deepfake corpus, presented in Table 3. This table displayed the list of lemmas characterizing each cluster. Each theme summarizes a unique facet of the discourse, providing insights into different perspectives, concerns, and narratives in the discussions on Quora. The following sections describe these themes, revealing the rich layers of meaning embedded within the deepfake discourse on Quora.

**Table 3:** The eight identified themes.

	**Theme 1:** **Deep fake generation technique**	**Theme 2:** **Detecting Deep fake anomalies**	**Theme 3:** **Deep fake creation apps**	**Theme 4:** **Ethical reflection and responsible use**
total occurrence (%)	71 (15.88)	54 (12.08)	23 (5.15)	65 (14.54)
lemmas (occurrence)	deep_learning (29)	able_to (21)	app (19)	potential (28)
	fake (62)	light (13)	google (6)	software (17)
	generator (17)	sign (17)	swap (9)	danger (14)
	discriminator (14)	shadow (11)	easy (8)	misinformation (14)
	technique (19)	blurry (9)	offer (5)	important (14)
	clip (16)	background (11)	play (5)	technology (41)
	base (12)	edge (8)	China (3)	use_to (30)
	portmanteau (7)	skin (7)	facial (11)	purpose (14)
	create (48)	tooth (7)	feature (8)	spread (15(
	hoax (6)	original (9)	photo (5)	responsibly (5)
	network (7)	perfectly (9)	expression (7)	create (41)
	refer (8)	noise (6)	look (14)	privacy (7)
	synthesis (8)	strange (7)	result (5)	risk (7)
	image (36)	inconsistency (9)	great (4)	associate (4)
	alter (11)	absence (6)	allow (5)	hashtags (4)
	term (12)	glitch (4)	spot (5)	job (4)
	synthetic_media (8)	inconsistent (5)	creation (2)	malicious (8)
	generative_adversarial_network (4)	telltale (5)	color (2)	manipulate (10)
	revenge (6)	double (4)	man (2)	increasingly (6)
	celebrity (16)	artifact (4)	moment (2)	fingerprint (4)
	**Theme 5:** **Entertainment and technological innovation**	**Theme 6:** **Threats to information integrity and societal impact**	**Theme 7:** **Facial mapping and superimposition**	**Theme 8:** **Facial cues and unnatural movements**
total occurrence (%)	79 (17.67)	63 (14.09)	18 (4.03)	74 (16.55)
lemmas (occurrence)	actor (20)	information (12)	target 89)	eye (19)
	film (20)	election (10)	model (13)	emotion (9)
	movie (17)	false (16)	lip (7)	lack (7)
	entertainment (14)	reputation (6)	train (59	talk (10)
	tid (9)	believe (17)	closely (3)	facial (20)
	star (8)	impact (6)	feature (8)	point (9)
	think (17)	political (9)	syncing (3)	natural (8)
	social (9)	threat (7)	zoom (4)	movement (14)
	medium (16)	democracy (4)	person (12)	pay (8)
	famous (7)	candidate (4)	common (3)	awkward (5)
	industry (8)	serious (5)	superimpose (3)	somebody (5)
	ban (5)	people (24)	capture (2)	attention (8)
	replace (12)	harm (6)	practice (2)	replicate (4)
	mimicry (4)	world (7)	screen (2)	position (6)
	queen_elizabeth (4)	reality (5)	slow_down (2)	facts (5)
	community (6)	fool (6)	training (3)	skeptical (5)
	tv (6)	damage (4)	facial (7)	sound (5)
	viewer (6)	mass (4)	expression (5)	blink (7)
	artificial_intelligence-generated (5)	truth (6)	latent (2)	focus (5)
	message (5)	cause (5)	data (4)	body (10)

*Theme 1*: *Deep Fake Generation Techniques*

This theme focuses on the techniques and technologies of creating deep fakes, particularly pinpointing deep learning and generative adversarial networks (GANs). The lemmas highlight crucial elements such as deep_learning, fake, generator, discriminator, technique, clip, and base. The context emphasizes the process of deep fake creation, where a generator produces fake video clips, and a discriminator distinguishes between real and fake content. The theme also underlines the possible implications of deep fake technology, including its misuse in generating fake celebrity content, revenge porn, and fake news. Overall, the theme provides insights into the mechanics of deep fake generation and its impact on media synthesis and manipulation. An example evidencing the public discussion about technical GAN technologies related to deepfake Is reported in the following: *“*Basically, the **generator creates** a **fake** video **clip** and then asks the **discriminator** to determine whether the **clip** is real or **fake**. Each time the **discriminator** accurately identifies a video **clip** as **fake**, it gives the **generator** a clue about what not to do when **creating** the next **clip**. Together, the **generator** and **discriminator** form something called a **generative_adversarial_network*”*.**

*Theme 2*: *Detecting Deep Fake Anomalies*.

This theme is about identifying anomalies and irregularities in deep fake content. The lemmas focus on characteristics such as able_to, light, sign, shadow, blurry, background, edge, skin, tooth, original, perfectly, noise, strange, inconsistency, absence, glitch, inconsistent, telltale, double, and artifact. The context emphasizes various signs and indicators that may reveal the presence of a deep fake. These signs include strange lighting or shadows, blurry or distorted features, skin tone or teeth abnormalities, inconsistent noise or audio, and background inconsistencies. The theme highlights the importance of recognizing anomalies such as artifacts, glitches, or double edges in the visuals and the absence of blinking or unnatural facial expressions. It also addresses the role of critical thinking in evaluating videos and suggests reporting deep fakes to platforms with policies against them. Overall, the theme provides insights into the visual and auditory cues that may indicate content manipulation through deep fake techniques, asreported in the example: “Strange **lighting** or **shadows**: deep_fake can sometimes have **strange lighting** or **shadows**, as the artificial intelligence model may not be able to recreate the **lighting** conditions of the **original** video perfectly. **Artifacts** or **glitches**: deep_fake can sometimes have **artifacts** or **glitches**, as the intelligence model may not be **able to perfectly** blend the two images”.

*Theme 3*: *Deep Fake Creation Apps*.

In the following example “Reface: ✔✔✔Reface: Face **Swap** artificial_intelligence **Photo App**–- **Apps** on **Google** PlayCreate & share **fun** face-swapping videos in seconds with amazing artificial_intelligence technology.// **play**. **Google**. com/store/**apps**/details? id = video. reface. app&hl = en&gl = USReface is another **great** deep_fake **app** that **offers** an impressive **feature** set*”* we note howthis theme focuses on applications used for creating deep fake content and the associated concerns, with lemmas like the app, google, swap, easy, offer, play, China, facial, feature, photo, expression, look, result, great, allow spot, creation, color, man, and moment. The primary context describes different apps on platforms like Google Play and Apple, offering features like face swapping, artificial intelligence-driven facial expression analysis, and creating realistic deep fake content from images and videos.

*Theme 4*: *Ethical Reflection and Responsible Use*.

This theme underlines the possible ethical dimensions and responsible application of deepfake technology. The key lemmas include “potential,” “software,” “danger,” “misinformation,” and “technology.” Discussions focus on the dual nature of deep fake technology, recognizing its potential for both positive and negative purposes. Concerns are raised about using deep fakes to spread misinformation, and users are advised to be aware of the associated risks. Tips for responsible use are provided, emphasizing transparency and protecting privacy. The sophistication of deep fake software and its evolving nature are underlined by stressing the relevance of increasing public awareness about deepfake. Overall, the theme emphasizes the need for a balanced understanding of the risks and benefits associated with deep fake technology, as we can see in the example: “This raises concerns about the **potential** for deep_fake to be used to **spread misinformation** or to **manipulate** people. It is **essential** to be aware of the **potential dangers** of deep_fake **software** and to use it **responsibly**. If you are considering using deep_fake **software**, it is **crucial** to be mindful of the ethical implications of this technology*”*.

*Theme 5*: *Entertainment and Technological Innovation*

This theme underlines the impact of deep fake technology on the entertainment industry and society. Key lemmas include “actor,” “film,” “movie,” “entertainment,” and “TID.” Discussions highlight the transformative possibilities of deep fake in the movie industry, envisioning scenarios where any actor can be seamlessly replaced or chosen post-production. Examples are provided, such as the recreation of iconic movie scenes with different actors. TID (The Indian deep_faker) is introduced as a prominent player in creating artificial intelligence-generated content for social media. The ethical implications of deep fake in entertainment are acknowledged, with perspectives on its positive and negative aspects. Overall, the theme emphasizes the evolving role of deep fake in shaping the entertainment landscape and its broader societal implications as we can see in the example: ✔ Not only can any **actor** be **replaced** in any past **film**, but **films** can be planned and produced in the future to replace the **actor**. ✔ Imagine a new **movie** experience, in which the **film** was shot and edited with any unknown **actor**, but you, the **viewer**, can **choose** later who you want to **star** in the **movie**.

*Theme 6*: *Threats to Information Integrity and Societal Impact*.

This theme revolves around the potential threats of deep fake technology to information integrity, elections, and societal stability. Key lemmas include “information,” “election,” “false,” “reputation,” and “believe.” Discussions emphasize the profound impact deep fake videos could have on elections, public opinion, and the economy. Concerns about the harm to reputations and the use of deep fake news to spread fake news, especially in the context of political manipulation, are highlighted. The narrative touches on the broader societal implications, discussing the erosion of trust in information sources and the challenges of differentiating between reality and manipulated content. The theme also acknowledges the dual nature of deep fake technology, which can be used for both positive and nefarious purposes, with a call for vigilance and awareness. This emerges indeed in the example: “I **believe** most of them present whatever **information** that supports their **political** narrative. There is plenty of MSMEDIA parroting the same **information** repeatedly. Much of it was **false**. Advertising has proven that the **masses** are affected positively by advertising. Most **people** I_ll **believe** the half truths of advertisements*”*.

*Theme 7*: *Facial Mapping and Superimposition*. This theme revolves around facial mapping and superimposition facilitated by deepfake technology. Key lemmas include “target,” “model,” “lip,” “train,” “feature,” and “syncing.” Discussions highlight the feature extraction process from a targeted individua’’s face, emphasizing the importance of capturing facial expressions, head movements, and speech patterns. Terms like “feature,” “syncing,” and “superimpose” highlight the focus on replicating facial expressions and movements. The elementary contexts describe how AI models, once adequately trained, can map the features of a target person onto different videos or images, achieving a realistic superimposition of faces onto various scenarios. The discussions emphasize the importance of feature extraction and the challenges related to unnatural lip-syncing and facial expressions in deepfake videos. The theme also discusses practical aspects, including the need for data to train the model and standard practices in deep fake creation. There is also a nod to the advancements in detection techniques and the importance of vigilance in identifying signs of deep fakes. This is for instance shown, in the xample: *“*Feature **Extraction**: The artificial intelligence model learns to extract key **features** from the **target** person’s face, such as facial expressions, head movements, and speech patterns. These features are crucial for ensuring that the generated content **closely** mimics the target’s behavior*”*.

*Theme 8*: *Facial Cues and Unnatural Movements*.

The eighth theme delves into the intricate details of facial expressions and movements in the context of deepfake technology. Key lemmas such as “eye,” “emotion,” and “lack” underscore the focus on replicating natural facial cues. The elementary contexts highlight the challenges in replicating authentic eye movements and facial expressions and the significance of paying attention to specific facial features such as cheeks, forehead, and eyebrows. Discussions emphasize the difficulty in mimicking natural blinking and eye movements, pointing out these challenges as potential red flags for detecting deepfake content. Common signs of deep fake videos are explored, including unnatural facial expressions, awkward facial positioning, and inconsistent body movement. The theme also addresses the challenges of replicating specific actions, such as blinking, in a natural way. Techniques for spotting facial morphing or image stitches are discussed, focusing on identifying emotions that may not align with the spoken content. The importance of scrutinizing facial feature positioning and looking for signs of a lack of emotion in videos is emphasized.

Overall, as shown in the example: “**Pay attention** to the **face**. . . . **Pay attention** to the cheeks and forehead. . . . **Pay attention** to the **eyes** and eyebrows. . . . **Pay attention** to the glasses. . . . **Pay attention** to the **facial** hair or **lack** thereof. . . . **Pay attention** to **facial** moles*”* this theme guides individuals to detect potential deep fake videos by examining specific visual and emotional cues.

#### Associations between cluster membership and publication date

**Fig 2:**
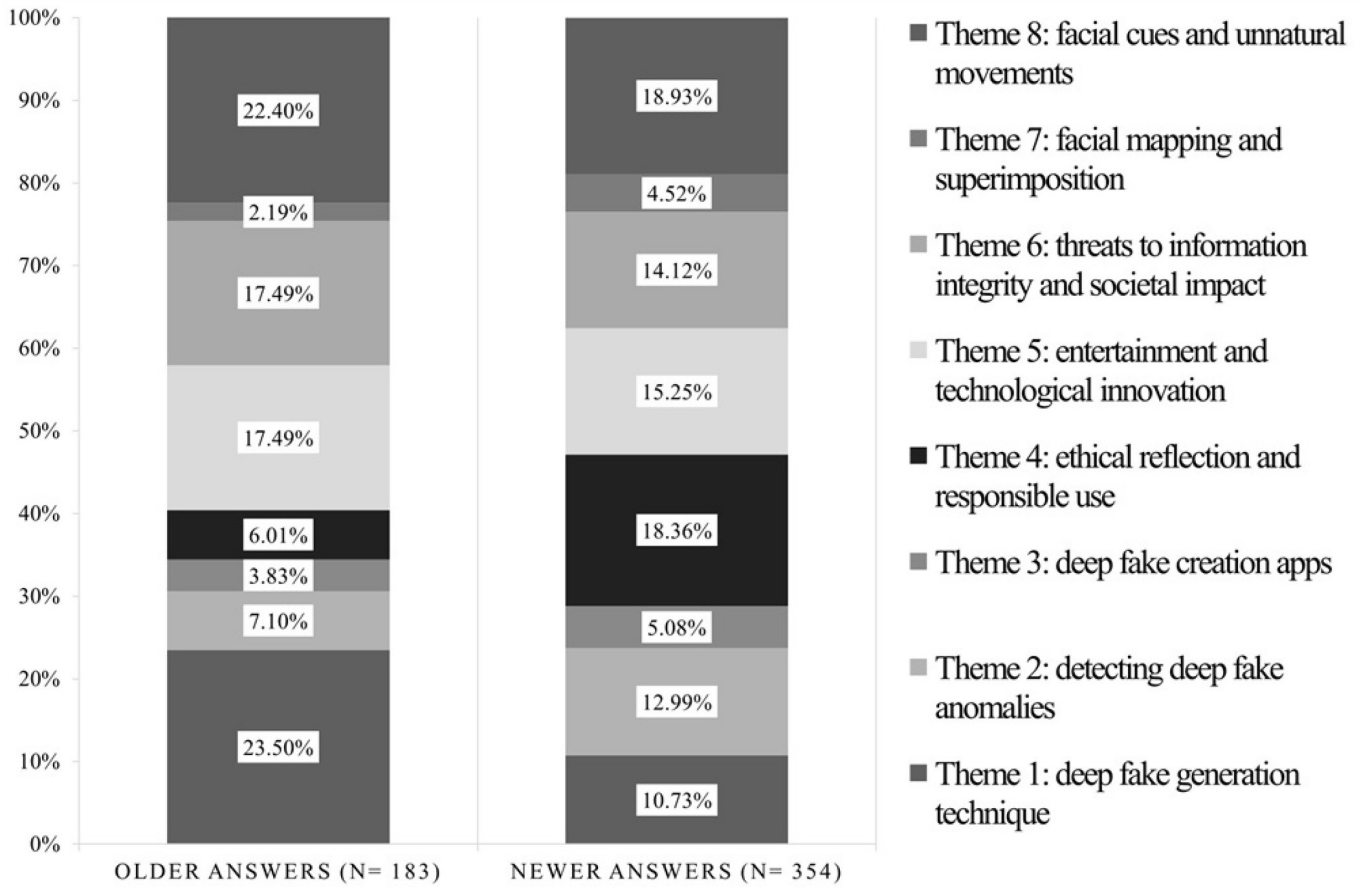
Percentages of elementary contexts classified into each cluster for older and newer answers.

The result of the Chi-square test highlighted significant associations between themes and publication date (χ^2^(7) = 34.01, p < .001). Specifically, as displayed in Fig 2, the first theme is more likely in older answers (SR = 3.92). In contrast, the second and the fourth themes are more likely in more recent answers–i.e., SR = 2.07 and SR = 3.89, respectively.

## Discussion

The current study investigated people’s mental representation of deepfake and its related contents in the human-computer interaction framework, applying a thematic analysis to public discussion on Quora online forums. The qualitative examination of discussions through Quora provided insights into how individuals perceive and engage with deepfake content in an online forum setting. This approach facilitated exploring the diverse themes of responses to deepfakes, revealing users’ expectations, concerns, and attitudes toward synthetic media.

Results evidenced eight main themes that differ in their specific contents and a temporal frame effect, showing significant differences between older and recent discussions around each theme. Public discussions on Quora focusing on Theme 1 ‐ Deep Fake Generation Techniques decreased from 25% to 11% from past to present, indicating that people have become more aware about the technical development of deepfake technology. Nowadays, creating artificial visual media has become remarkably easy. The rising popularity of deepfakes is due to their convincingly realistic videos and user-friendly interface, which are accessible to individuals with diverse levels of computer proficiency. Generating convincing fake videos is becoming progressively simpler due to technical advancements in AI. Now, all it takes is a target person’s photo or a brief video to produce remarkably realistic altered content [76]. Similarly, discussions on Theme 2 –Detecting Deep Fake Anomalies, which collects people’s opinions about media inconsistencies and irregularities in deepfake data, increased from 10% to 14% from older to recent times. The primary emphasis in people’s discussions is on strategies and methods for deepfake detection, which is focused on identifying anomalies to detect artifacts and traces originating from the underlying AI generative process [77]. In deepfake detection, visual cues remain valuable, orienting people’s ability to detect real vs. fake media, but determining the authenticity of a video goes beyond just visual processing. From a psychological perspective, we could argue that deepfake detection involves considering both the technological context and critical thinking skills, so adapting beliefs based on new information [38,78].

The prevalence of discussions related to Theme 3 *Deep Fake Creation Apps* varied from 3% in old posts to 6% in recent ones, suggesting a growing interest in applications associated with deepfake technology. Characterized by the ability to manipulate and generate realistic audiovisual content, deepfake applications inherently possess a novelty factor. Recent studies on media technologies suggest that individuals could be initially drawn to the uniqueness and creativity associated with deepfakes, perceiving them as a novel form of entertainment [79]. In this sense, deepfakes could enhance the well-known *novelty effect* in people, defined in psychology as a phenomenon that describes the initial surge of interest and attention individuals show when exposed to something new or novel. Mainstream psychology used it in the context of research related to human perception [80] elving into the role of curiosity and arousal and exploring how novelty can contribute to increased interest and attention. Besides, the novelty effect was related to learning and behavior. Hull’s work on habit-family hierarchy and maze learning touches upon the role of novel stimuli in influencing learning and behavior [81]. The novelty effect has also been associated with dispositional traits such as curiosity [82] and sensation-seeking [83]. Regarding media psychology, the novelty effect has been observed in various technological advancements, influencing the perception and adoption of novel forms of media and entertainment [29,84], affecting whether they are seen as a source of entertainment or perceived as a potential threat.

Regarding Theme 4: *Ethical Reflection and Responsible Use*, results show that discussions significantly increased from 5% in older discussions to 19% in recent ones, indicating a heightened awareness and discussion of ethical implications surrounding deepfake technology. Deepfakes are exploited as tools for unethical practices in fields such as pornography [85,86]. Moreover, scholars highlighted the dangers and harms related to deepfakes [11, Although deepfakes have received recently academic attention it is increasingly evident that they offer benefits and opportunities in social and medical fields (i.e., assisting individuals with Alzheimer’s disease in interacting with a younger face they may recall). In 2020, a deepfake video revived a victim of the Parkland school shooting in 2018, advocating for gun safety legislation [87] (Diaz, 2020). This highlighted the potential of deepfakes for promoting pro-social causes. Evidence of positive Deepfake technology use is scarce, but it has been demonstrated to have the potential for positive employmentImplementing deepfakes in *FakeForward*, which refers to models involving peers, hasdemonstrated that desirable behaviors and skills are encouraged, which increases performance and confidence, among others. It is assumed that when selecting video material to use with *FakeForward*, it is essential to choose content that showcases activities contributing to an individual’s positive development while ensuring protection from damage [88]. The current psychological literature evidences the framing effect, which relies on a strict interdependency between how technology is portrayed in the media and people’s mental representations and beliefs about it [89]. The framing effect could influence cognitive processing, emphasizing the positive aspects associated with the novelty of deepfakes. As discussed, deepfakes can potentially foster trust and prepare individuals for the digital era. It could bolster collective critical thinking, mitigate susceptibility to misinformation, and encourage rigorous source verification. This, in turn, facilitates a purposeful transition from instrumental rationality to a socially informed trust paradigm in the digital age [13].

Results about *Theme 5*: *Entertainment and Technological Innovation* evidenced that people’s interest in using deepfake for entertainment decreased slightly from 19% to 15%, suggesting a slight decrease in interest in the specific topic. Numerous Deepfake applications exhibit creativity, educational value, and amusement, yet they are often overlooked in the literature, focusing on the negative aspects [90]. Deepfakes have been suggested to personalize and make films, video games, and other media by incorporating one’s face onto characters. For instance, in the trailer for the film Gemini Man in 2019 starring Will Smith, deepfake technology was employed in an old clip of Will Smith from the television series The Fresh Prince of Bel-Air, in which he talked about the movie. They also can potentially supplant CGI (Computer-Generated Imagery) in the film industry [88]. This technology is often controversially employed to bring actors back to life. As in this case, research suggests that media framing is crucial in shaping individuals’ cognitive schemas regarding technology [66]. Different frames, such as innovation or risk frames, can influence how people acts. Iyengar (1991) In [91], it is discussed how media frames can activate existing cognitive schemas, influencing individuals’ interpretation of information, and, in turn, this interaction contributes to the formation of people’s beliefs and attitudes. Research examined the framing of technology in news media, arguing that positive framing tends to enhance beliefs in the benefits of technology, while negative framing emphasizes risks and potential drawbacks [92]. Recent examination of media framing in the context of AI underscores the role of media framing in shaping cognitive schemas related to the societal implications of AI [93]. Prior literature also focused on how media significantly influences public perception and adoption of new technologies. Positive portrayals in the media can create an optimistic view of technological advancements, fostering enthusiasm and willingness to adopt innovations. In contrast, negative portrayals in the media could negatively influence public mood and perceptions [45] (Chen, 2004). Then, we could also hypothesize that deepfakes could be more likely interpreted by people as a novel and creative form of content rather than a potential threat when presented in an entertainment framing.

Moreover, the results of the current thematic analysis also evidenced *Theme 6 ‐ Threats to Information Integrity and Societal Impact*, which collects discussions about *potential* threats of deepfake, evidencing an increasing trend from 9% in older discussions to 14% in recent ones, so indicating a growing concern people have regarding the potential misuse or threats posed by deepfake technology. As advanced tools for crafting misleading narratives, Deepfakes pose a significant risk of spreading false information. This perpetuation of falsehoods can prompt individuals to propagate rumors unknowingly or even intentionally, contributing to the spread of misinformation. The influence of misinformation on melding political perspectives, as the emergence of deepfakes carries significant implications for shaping political convictions and amplifying societal divisions [94]. Misinformation manifests in various forms, from isolated audio clips to fake news and low-quality manipulated media, such as cheap fakes [19]. Anyway, the deleterious impact of social media on democratic processes surpasses conventional misinformation [11]. Take, for instance, the emerging prevalence of deepfakes, which pose a significant and hostile threat, potentially leading to an "information apocalypse." In such a scenario, distinguishing between fact and fiction becomes increasingly challenging for citizens [11]. However, when individuals become aware of the falsehoods or face social repercussions for spreading them, they may seek to distance themselves from the rumors or halt their involvement in their propagation [95]. Deepfakes can be a potent tool for creating false narratives, making it imperative to study how exposure to such content influences the formation of false beliefs. From this point of view, deepfakes have the potential to shape political beliefs and exacerbate social polarization [95]. The role of misinformation in influencing political attitudes, understanding how deepfakes contribute to these dynamics is essential for comprehending the broader societal impact on political discourse and social cohesion].

Also, results on *Theme 7*: *Facial Mapping and Superimposition* evidenced an enhancement in discussions about facial mapping from 1% to 3% in more recent discussions, suggesting an imperceptible growing interest in the technical aspects of facial manipulation. However, it is noteworthy that discussions about *Theme 8*: *Facial Cues and Unnatural Movements* have decreased from 19% to 12% during the same period. This appears to be a shift towards a greater interest in the technical aspects of facial manipulation rather than the analysis of facial expressions themselves as well as tools for face swapping improved over the time. In this context, the challenges lie in accurately reproducing genuine eye movements and facial expressions. It underscores the necessity of paying attention to specific facial features such as cheeks, forehead, and eyebrows, as well as the challenge of mimicking natural eye movements, citing these difficulties as potential red flags for identifying deepfake content. Moreover, as emerged from recent studies [96], understanding users’ eye movements and reactions to deepfake content is crucial for enhancing the realism and human-likeness of deepfakes, which can impact user trust and engagement. Indeed, a key precursor to lead people to accept the potential benefits of deepfake is overcoming the so-called uncanny valley effect [97]. This effect usually brings people to experience a feeling of discomfort when humanoid robots are highly realistic [96].

### Strengths and limitations

Overall, this study has several strengths. First, it represents the first attempt to systematically identify and figure out themes related to deepfake content perception. Our qualitative approach allowed for an in-depth exploration of cognitive mental representations involved in people’s perception of deepfake content. Specifically, this study contributes to the literature by comprehensively exploring the discourse surrounding deepfake technology. This work provides insights into the most frequent lemmas, semantic relationships, and themes related to this relevant topic through co-occurrence analysis and semantic clustering. This approach allowed for a better understanding of the diverse perspectives and narratives surrounding deepfake technology. Furthermore, by testing the associations of thematic clusters with answer publication dates, we gained insights into the temporal dynamics of deepfake-related discourse, highlighting how cognitive representations of deepfake might have evolved.

However, it is essential to acknowledge some limitations inherent in this study. Firstly, our approach is solely qualitative and does not incorporate quantitative data, limiting the depth of our analysis and the generalizability of our findings. Secondly, one potential limitation is the size of the text corpus analyzed. The final corpus consisted of 166 answers to 17 questions. While this may seem small, it is in line with recommendations for thematic analysis, as 100–200 items is a sufficient sample size for a small-scale thematic analysis project [98]. Given the exploratory nature of this study and the emerging nature of the deepfake phenomenon, the sample size is appropriate for the research objectives. It is important to note that it was not possible to increase the sample size beyond the 166 answers, as this represented the total number of relevant questions and answers available on the Quora platform at the time of data collection. Future research could expand on these findings by analyzing a larger corpus of data from different sources. Additionally, using Quora data might lead to potential biases in online platforms, such as the lack of demographic information about the users contributing to the discussions. Furthermore, we should also consider that users who contribute to discussions on Quora about deepfake technology may not represent a random population sample. Thus, while our study offers valuable insights into the cognitive processes underlying deepfake perception, the generalizability of its findings may be limited to Quora’s users and may not fully capture the variety of perspectives on this topic.

In summary, while this study offers valuable insights into the complex cognitive representations of deepfake, careful consideration of these strengths and limitations is essential for interpreting, contextualizing, and generalizing our findings.

## Conclusions

Studying the relationship between deepfakes and human mental representations helps develop practical media literacy programs. Individuals with higher media literacy are more resistant to misinformation [99]. Media literacy involves critically understanding and analysing both traditional and new media messages, including the ability to access, evaluate, and create media content, while comprehending its societal impact [42]. This empowers users to engage thoughtfully with media content, while also enabling them to identify biases. Therefore, future studies should delve deeper into media literacy to enhance critical thinking and resilience against manipulative content. People could also have negative depictions in media that may lead to skepticism, fear, or resistance [100]. From this point of view, media often create cognitive dissonance [101] when public expectations clash with the reality of technological developments. Studies suggest that discrepancies between media portrayals and actual technological outcomes can lead to cognitive dissonance, influencing individuals to reevaluate their beliefs and mental representations of technology [102]. As deepfakes can particularly influence memory formation and recall, future research perspectives could explore how prolonged exposure to deepfakes may influence individuals’ memory, beliefs, and behavior over time, changes that could result from interaction with manipulated media content. Research indicates that exposure to misinformation, including deepfakes, can affect memory and the perceived accuracy of information [103]. Studying how deepfakes impact cognitive processes is essential for comprehending the potential distortion of individuals’ beliefs and memories. Furthermore, deepfakes pose a challenge to trust in media and information sources. Studies demonstrate that the perceived quality of information influences perceived trust in media, and deepfakes can manipulate this perception [29]. Examining the impact of deepfakes on trust is crucial for developing strategies to mitigate potential harm to the credibility of media and information sources. These studies highlighted the need to educate the public about the potential impact of deepfakes and the importance of critical thinking skills in discerning their authenticity. Additionally, efforts from various stakeholders, such as platforms, journalists, and policymakers, are necessary to counteract the adverse effects of deepfakes.

As the psychological literature on deepfakes grows, researchers and policymakers are actively exploring mitigation strategies. These may include education campaigns to enhance media literacy, developing robust detection algorithms, and the implementation of regulatory frameworks. Future research directions should focus on understanding the long-term effects of deepfake exposure, exploring individual differences in susceptibility, and refining interventions to foster critical thinking in the face of synthetic media. This thorough examination of the psychological literature on deepfakes underscores the multifaceted impact of this technology on individuals’ beliefs and cognitive processes. Researching factors that contribute to varying vulnerability to deepfakes involves exploring aspects such as age, level of digital literacy, previous encounters with misinformation, and personal psychological traits. These elements not only affect susceptibility to deepfake content but also influence interactions between human perceptions and technology. As advancements in deepfake technology persist, grasping its psychological implications becomes imperative for formulating effective measures to alleviate possible harm and cultivate a robust and knowledgeable community.

## Supporting information

S1 File List of URLs of the 17 considered quora questions. (DOCX)
