# Supplementary material for: The public mental representations of deepfake technology: An in-depth qualitative exploration through Quora text data analysis
Source: PLoS One. 2024 Dec 30;19(12):e0313605. doi: 10.1371/journal.pone.0313605 (PMC11684586; doi:10.1371/journal.pone.0313605)
Supplement: S1 File — (DOCX) [file pone.0313605.s001.docx]

**Supplementary file. List of URLs of the 17 considered Quora questions**

1. https://www.quora.com/Are-deepfake-videos-really-a-problem
2. https://www.quora.com/Considering-the-kinds-of-speech-the-US-Supreme-Court-has-determined-are-not-protected-by-the-1st-Amendment-in-what-ways-can-Deep-Fakes-be-regulated-and-or-banned-on-social-media
3. https://www.quora.com/Did-Channel-4-s-Alternative-Christmas-Message-2020-delivered-by-a-deepfake-Queen-Elizabeth-II-go-too-far-Why
4. https://www.quora.com/Do-you-think-there-are-some-benefits-to-DeepFake
5. https://www.quora.com/Do-you-think-youd-be-able-to-spot-a-Deepfake
6. https://www.quora.com/Have-any-news-media-corporations-attempted-to-use-deepfake-or-deepfake-like-videos-or-images-to-deceive-their-audience
7. https://www.quora.com/How-do-Deep-Fakes-work
8. https://www.quora.com/What-are-some-telltale-signs-of-a-deepfake
9. https://www.quora.com/What-are-the-dangers-of-deepfakes
10. https://www.quora.com/What-do-you-think-of-Deepfakes
11. https://www.quora.com/What-is-behind-the-popularity-of-Deepfake-despite-the-fears-of-misinformation
12. https://www.quora.com/What-is-deep-fake-AI
13. https://www.quora.com/What-is-deepfake-software
14. https://www.quora.com/What-is-Deepfake-technology
15. https://www.quora.com/What-is-deepfake-technology-What-are-the-threats-posed-by-deepfakes
16. https://www.quora.com/What-is-deep-fake-video
17. https://www.quora.com/What-resources-are-available-to-identify-deep-fakes
